# Prenatal Developmental Toxicity and Histopathological Changes of the Placenta Induced by *Syzygium guineense* Leaf Extract in Rats

**DOI:** 10.1155/2022/5209136

**Published:** 2022-10-11

**Authors:** Melese Shenkut Abebe, Kaleab Asres, Yonas Bekuretsion, Samuel Woldekidan, Bihonegn Sisay, Girma Seyoum

**Affiliations:** ^1^Department of Anatomy, School of Medicine, College of Medicine and Health Science, Wollo University, Dessie, Ethiopia; ^2^Department Pharmaceutical Chemistry and Pharmacognosy, College of Health Sciences, Addis Ababa University, Addis Ababa, Ethiopia; ^3^Department of Pathology, College of Health Sciences, Addis Ababa University, Addis Ababa, Ethiopia; ^4^Traditional and Modern Medicine Research Directorate, Ethiopian Public Health Institute, Addis Ababa, Ethiopia; ^5^Department of Anatomy, College of Health Sciences, Addis Ababa University, Addis Ababa, Ethiopia

## Abstract

Many of the traditional herbal products are served to the consumer without proper efficacy and safety investigations. A laboratory-based experimental study was employed to investigate the toxic effects of *Syzygium guineense* leaf extract on the fetal development and histopathology of the placenta in rats. Fifty pregnant Wistar albino rats were randomly allocated into five groups, each consisting of 10 rats. *S. guineense* leaf extract, at doses of 250, 500, and 1000 mg/kg of body weight, was respectively administered to groups I-III rats. Groups four and five were control and *ad libitum* control, respectively. The number of resorptions, implantation sites, and live or dead fetuses was counted. The weight and crown-rump length of the fetuses were measured. The histopathological investigation of the placenta was conducted. Administration of 70% ethanol extract of *S. guineense* leaves reduced weight gain and food intake of pregnant rats at *p* value <0.05. The crown-rump length of the near-term rat fetus was significantly reduced in rats treated with 1000 mg/kg body weight of *S. guineense* extract (*p* value <0.05). The plant extract did not affect the number of implantations, fetal resorptions, live births, and stillbirths. The weight of the fetuses and the placentae also decreased dose-dependently. Decidual cystic degeneration was the most prevalent histopathological change observed in a rat's placenta treated with 1000 mg/kg body weight of *S. guineense* extract. Consumption of *S. guineense* leaves, especially at a high dose, may affect fetal development. Therefore, liberal use of *S. guineense* leaves during pregnancy should be avoided.

## 1. Introduction

*Syzygium guineense* wall. is a commonly used medicinal plant in many African countries, including Ethiopia, Eritrea, and Somalia [1]. Researchers reported that the plant has demonstrated efficacy against hypertension [2], hyperglycemia [3], cancer [4], pain and inflammation [5], and many others. The effectiveness of *S. guineense* against a variety of illnesses is credited to its phytochemical components. Polyphenols, essential oils, alkaloids, terpenoids, anthraquinones, flavonoids, tannins, saponins, glycosides, and triterpenes are commonly identified secondary metabolites of the leaves of *S. guineense* [6, 7].

Many traditional herbal products are sold to consumers without proper efficacy and safety evaluations [8]. This activity has been reported in different countries. However, research should be conducted to investigate the noxious effects of herbal products antecedent to their use by consumers. Because various undesirable results, including death, have been reported from the use of herbal products [9, 10].

Prenatal exposure to chemicals may lead to abnormal development in the embryo/fetus [11, 12]. This can be manifested by the presence of malformed organs, developmental delay, functional deterioration, complete or partial agenesis of organs, and fetal death. Chemical exposure during pregnancy can directly affect fetal development independent of their toxicity to the mother [13]. Following maternal exposure to chemical agents, many of these chemicals can pass through the placental membrane and reach the embryo/fetus. These chemicals have the potential to disrupt the normal development of their offspring [14].

Toxicological screening of any potential drug for its effect on intrauterine development has become mandatory before use in humans. Investigating the hurtful effect of plant products on intrauterine development is one of the most pompous investigations to be performed [15]. In our previous study conducted to evaluate the effect of *S. guineense* on skeletal and soft tissue development, there was no significant developmental delay observed in the genital or other organs [16]. On the other hand, another study reported that consumption of *S. guineense* extract did not produce significant developmental anomalies in the reproductive organs, including anogenital distance and nipple retention [17]. The detailed effect of *S. guineense* on fetal development has not been determined yet. Therefore, our study was concerned with investigating the toxic effects of *S. guineense* on the fetal development and histopathology of the placenta in rats.

## 2. Materials and Methods

### 2.1. Collection and Extraction of the Plant Material

The leaves of *S. guineense* were harvested from the surroundings of Woliso town, 113 km away from Addis Ababa, the capital city of Ethiopia. The test plant was verified by a taxonomist in the National Herbarium of Ethiopia with a voucher sample (MS 001) deposited for later remark. Regarding the extraction procedure, we followed the methods of Abebe et al. [18]. Briefly, the leaves of the test plant were cleaned, shade dried, broken into pieces by hand, and roughly ground by an electric mill. For extraction, the powder of the dried leaves was macerated in 70% ethanol for 24 hours with frequent rotation by an orbital shaker. After that, the blend was refined by Whatman paper (No. 1, 18 cm in diameter). The blend was evaporated with a rotatory evaporator at 40°C and the remaining extract was further seared in a hot water bath at 45°C. The dried (solvent-free) extract was stored in a refrigerator at −4°C until administration to the rats [19].

### 2.2. Test Animals

Our test animals were Nulliparous Wistar albino rats, 220–240 g in weight, and aged 10–12 weeks. The experimental animals were purchased from the Ethiopian Public Health Institute (EPHI) animal rearing house. The test animals were adapted to the new laboratory conditions for five consecutive days. The rats were kept in a comfortable stainless-steel cage at room temperature (23 ± 3°C) with a relative humidity of 50 ± 10% under a controlled alternating 12-hour light-dark cycle. The experimental rats were fed a standard laboratory diet and served with drinking water unlimitedly.

### 2.3. Experimental Design

The rats were mated by placing a single female rat in a cage containing one unrelated male rat of proven fertility. Following an overnight mating period, the next morning, mated female rats were inspected for the presence of a copulatory plug. Thereupon, a vaginal smear test was conducted, and the presence of sperm in the vaginal smear was considered day one of pregnancy. This study employed three treatment and two control groups of rats. Fifty pregnant rats were randomly (using a computer-based random order generator) assigned into five groups, each consisting of 10 rats. The treatment groups (groups one, two, and three) were treated with 250, 500, and 1000 mg/kg of the *S. guineense* extract dissolved in distilled water via intragastric tube, respectively. The first control group (group IV) was given distilled water at 1 ml/100 g of body weight. This group was used to ascertain whether the outcomes were due to manipulation (stress) or not. The second control group (group V or *ad libitum* control or untouched control) was neither an extract nor distilled water treated and used as a standard comparison group. The treatment doses were selected based on a previous efficacy study report [3]. The duration of the treatment period was day-6 through day-12 of pregnancy, which is a critical phase of organ formation in rats. Throughout the treatment period, the food intake of pregnant dams was measured daily. Pregnant rats were weighed at the confirmation of pregnancy, beginning of treatment (6^th^ day of pregnancy), end of treatment (12^th^ day of pregnancy), and necropsy (20^th^ day of pregnancy). Weight gain at the time of gestation was computed and compared between the treatment and control groups. We followed the methods described by Seyoum and Persaud [20] and Seyoum [21].

On day 20 of gestation, the pregnant rats were anesthetized by an intraperitoneal injection of pentobarbital (150 mg/kg body weight) [22] and sacrificed humanely. The abdomen was opened by a longitudinal incision and the gravid uterus was kept intact, and the following evaluations were carried out. The number of implantation sites was counted. The number of fetal deaths and viable fetuses were counted by applying gentle pressure to the fetus. The number and degree of resorption (early or late) sites was counted by checking uterine nodules that were not occupied by living or recently dead fetuses. After the aforementioned examinations, the two horns of the uterus were incised along the antimesometrial border. Then, the fetuses, placentae, and associated fetal membranes were exposed. The placentae and fetal membranes were removed and measured separately. Fetal sex was identified as well as the weight and crown-rump length (CRL) of each fetus were recorded.

### 2.4. Examination of the Placenta

Each placenta was examined for any gross morphological abnormalities. Placentae of two-three fetuses/dam/group were selected for further histopathological examination. A 3–4 mm section was sampled from each placenta and immersed in 10% formalin for fixation. With regard to tissue processing and staining, we followed the methods of Abebe et al. [18]. Following an overnight fixation, the tissues were dehydrated by an ascending series of alcohol (40%, 50%, 70%, 80%, 90%, and 100%). The tissues were then cleared by xylene (I, II, and III). After clearing, the tissues were impregnated with melted paraffin wax (I and II). Finally, each sample tissue was placed in an embedding cassette and filled with melted wax. A five µm section was made for every block, and the ribbon was placed on the frosted slide and kept in a hot oven (40–45°C) for 20–30 minutes [23].

The staining of the tissues was based on the following procedures: the slides were dewaxed with three-steps xylene for five minutes in each, rehydrated with a descending series of alcohol (absolute alcohol I, absolute alcohol II, 90% alcohol, 80% alcohol, and 70% alcohol) for two minutes in each, washed with running tap water, followed by staining with Harris hematoxylin for 5–10 minutes, cleaned with running tap water for 10 minutes, immersed in acid alcohol for 2–3 seconds, and counterstained with eosin Y for 1–2 minutes. Once the slides were stained, they were subjected to an ascending series of alcohol (80%, 95%, absolute alcohol I and II) for dehydration and xylene for clearing. Finally, the cleared slides were mounted with Dibutylphthalate Polystyrene Xylene (DPX) and covered with a cover-slip [23].

In the stained slides, a senior pathologist investigated the structural integrity of the placenta using a binocular light microscope. The decidual zone, the labyrinthine zone, giant cells, and trophoblasts of the placenta were investigated, and important findings were photographed by an automated built-in digital microscope camera (Leica EC4, Germany) under 10× and 20× objective lens magnification.

### 2.5. Statistical Analysis

Data analysis were performed by a statistical package for social science (SPSS) version 24. The difference between groups was analyzed by one-way analysis of variance (ANOVA) followed by Turkey post-hoc test for multiple comparisons. Moreover, the frequency of placental histopathological changes was calculated and the difference between groups in the frequency of placental changes was checked by a chi-square test. Results are expressed as mean ± standard deviation of mean (SDM) and percentages. A cutoff point value for statistical significance was *p* value <0.05.

### 2.6. Ethical Approval

This research was conducted following an ethical approval letter obtained from the Institutional Review Board at the College of Health Sciences, Addis Ababa University. All protocols were conducted in obedience to the recommendations of the Organization for Economic Co-operation and Development (OECD) guidelines [24] and the highest standards of the laboratory of EPHI.

## 3. Results

### 3.1. Maternal Food Intake and Weight Gain

The levels of food intake are presented in Table 1. The high dose (1000 mg/kg) treated group consumed a significantly lower amount of food compared with the controls and low dose (250 mg/kg) treated groups. The weight gain of pregnant rats was measured between pregnancy days 6–12 and 13–20. All treated groups showed significantly reduced weight gain during the first 6–12 days of pregnancy, compared with the *ad libitum* control group. In addition, rats that received *S. guineense* extract at a 1000 mg/kg dose showed a significant weight gain reduction during days 13–20 of pregnancy as compared to the control and *ad libitum* control groups (Table 1).

### 3.2. Pregnancy Outcomes

**Figure 1:**
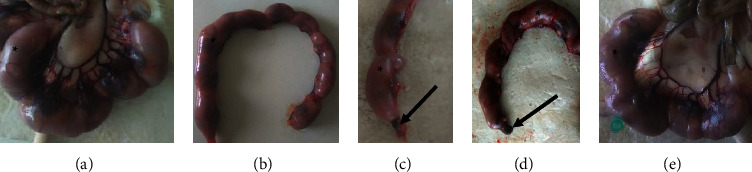
Gravid uterus of rat from group I, (a) group II, (b) group III, (c) group IV, and (d) group V (e) indicating implantation sites (*∗*) and fetal resorption (arrow) following administration of ethanol leaf extract of *S. guineense*.

In the current study, the pregnancy outcomes measured were the number of implantation sites, resorptions, live/dead fetuses, and sex of the fetus (Table 2). Treatment of pregnant rats with *S. guineense* extract did not significantly alter the average number of either implantation or resorption sites (Figure 1). In addition, no significant change in the number of live or dead fetuses was observed between the treatment and the control groups.

### 3.3. Fetal Growth

Regarding the fetal growth indices (CRL, fetal weight, and placental weight), a significant reduction of CRL was observed in fetuses treated with 1000 mg/kg body weight of the plant extract, compared with the control and *ad libitum* control groups. The CRL of fetuses in the high dose and *ad libitum* control groups was 5.0 ± 0.4 and 5.7 ± 0.4, respectively. The weight of the fetuses and placentae showed a dose-dependent reduction in the treatment groups. However, this was not significant when compared to the control or *ad libitum* control groups (Table 3).

### 3.4. Placental Histopathology

**Figure 2:**
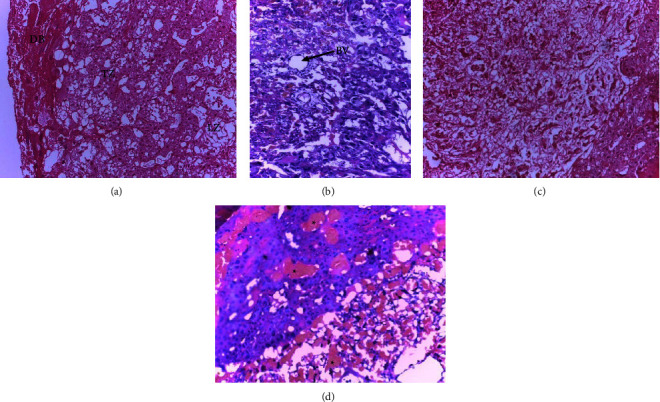
Photomicrograph of rat placenta of group IV, (a) group V, and (b) group I (c) showing normal architecture of the placenta and group II (d) showing hematoma in the different zones of the placenta (*∗*); DB: Decidua basalis, TZ: Trophoblastic zone, LZ: Labyrinthine zone, BV: Blood vessel; H and E stain, 100× total magnification.

**Figure 3:**
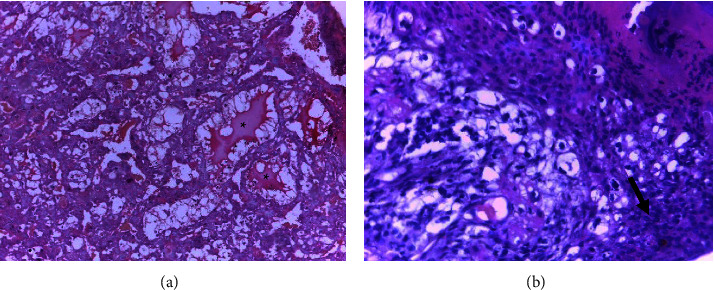
Photomicrograph of rat placenta (a & b) from a rat treated with 1000 mg/kg of ethanol extract of *S. guineense* leaves (group III) showing decidual cystic degeneration (*∗*) and trophoblast proliferation (arrow); H and E stain, 200× total magnification.

Microscopic examination of the placenta showed some structural changes in the decidual and labyrinthine zones of the placenta (Figures 2 & 3). Decidual cystic degeneration, hemorrhage, and trophoblast proliferation were the histopathological changes observed. Although the frequency of decidual cystic degeneration was higher in rats treated with a high dose of the plant extract, none of the above changes were statistically significant (Table 4).

## 4. Discussion

Prenatal development can be divided into three phases: pre-embryonic, embryonic, and fetal periods [25]. The embryonic period, day 6–12 of gestation in rats, is a critical period where organs of the embryo can be damaged if exposed to a teratogen [21]. In intrauterine life, the developing fetus is not fully protected from toxicants. Studies have reported that many environmental chemicals can pass through the placental membrane, and as many as 200 foreign chemicals have been detected in the blood samples taken from the umbilical cord [26]. Due to the undeveloped metabolic function of the liver and the excretion capacity of the kidney, the level of toxicants in the fetal circulation is much greater than in the maternal circulation [27]. Toxic agents can directly or indirectly affect embryos/fetuses. When the toxic agents cross the placental membrane, they directly damage the developing embryonic/fetal tissue. Indirectly, toxicants that damage the placental tissue and compromise placental function might impede the development of embryos/fetuses [28].

The objective of the present study was to evaluate the toxic effects of prenatal exposure of *S. guineense* on the fetal development and histopathology of the placenta. In the present study, administration of the 70% ethanol extract *S. guineense* to the pregnant rats during the crucial period of organogenesis (day-6 through day-12 of gestation) resulted in reduced food intake and weight gain of the pregnant dams and CRL of 20-day old rat fetuses in the high dose (1000 mg/kg body weight) treated group.

Maternal weight gain during the treatment period was affected by the administration of the plant extract despite similar intake of food. It was witnessed by a significant and dose-dependent reduction in weight gain in the rats treated with higher doses of the test plant. Reduction in weight gain was also reported by other researchers: Abba et al. [29], Loha et al. [30], and Amare [31], who conducted toxicity studies on nonpregnant rats and mice following administration of *S. guineense* extract. Moreover, the food intake of pregnant rats in the treatment group decreased significantly compared to the control groups. A study conducted by Rogers and Kavlock [32] reported that decreased food intake can induce weight loss and other clinical signs. Therefore, this could be the reason for the decrease in weight gain during gestation. As reported by Chung et al. [33], tannins damage the epithelial coverings of the digestive tract and reduce food intake. *S. guineense* is a tannin-rich plant [34, 35], and the presence of tannins in high concentrations may explain the reduced food intake in the *S. guineense* treated rats.

Retarded fetal development *in vivo* is manifested by decreased fetal weight, placental weight, and CRL [36]. In the current experiment, treatment with 1000 mg/kg of *S. guineense* extract significantly reduced the CRL of the fetuses when compared with the control groups. In addition, there was a reduction in fetal and placental weights. This finding is in line with the previous study [16]. The leaves of *S. guineense* possess secondary metabolites such as terpenoids, anthraquinones, flavonoids, tannins, saponins, glycosides, triterpenes, and phenols that pass through the placental membrane and hurt fetal development [7, 37, 38]. These secondary metabolites of *S. guineense* might be responsible for the decrease in the CRL of the treated rat fetuses [16].

The plant extract had no effect on the other pregnancy outcomes, including the number of implantations, fetal resorptions, live births, and stillbirths. This refers that the test plant may not have a significant detrimental effect on the progression of pregnancy in rats.

The placenta plays a central role in the transfer of nutrients, gases, metabolic wastes, drugs, and immunoglobulins between the mother and the embryo/fetus. It also allows the passage of toxicants, mycotoxins, plant alkaloids, and many others [39–42]. Due to its function, the placenta is highly sensitive to toxins [39]. Succeeding maternal exposure to chemicals, many of these chemicals can pass through the placental membrane and reach the embryo/fetus. Thus, the chemicals can affect the fetus, the placenta, and even the mother herself [14]. Administration of chemical agents in the early embryonic period, since the trophoblast cell differentiation is not complete to make the placental membrane [43], can affect the development of embryonic tissue and the placenta [44].

In the present study, decidual cystic degeneration, hemorrhage, and trophoblast proliferation were seen in both treatment and control groups. A greater frequency of decidual cystic degeneration was prevalent in rats treated with 1000 mg/kg body weight of *S. guineense*. However, these alterations in the microscopic structures of the placenta were not statistically significant. Similarly, in another study, there were no significant histological alterations observed in the liver and kidney of rats treated with *S. guineense* extract [18]. The decidua basalis is found at the base of the placenta and it contains newly formed vasculatures. The decidua basalis mainly develops during early pregnancy, and it undergoes regression after gestational day 11 in rats. As a result, decidua is less sensitive to chemical exposure in the embryogenesis period than the other parts of the placenta. Moreover, hemorrhage can be seen when decidual regression occurs [45]. This might be the justification for the presence of decidual cystic degeneration and hemorrhage in both treated and control groups. On the other hand, spongiotrophoblasts, glycogen cells, and trophoblastic giant cells were not noticeably affected by treatment with the plant extract.

## 5. Limitations of the Study

The current study provided evidence regarding the developmental toxicity of *S. guineense*, which contributes to the knowledge of science in terms of the developmental toxicity of *S. guineense*. However, it is not without limitations. The main limitation is that, due to financial constraints, advanced tests such as immunohistochemistry and electron microscopy were not included. Secondly, the test substance was administered only from day 6–12 of gestation. It would have had value if the treatment period was the whole period of pregnancy.

## 6. Conclusion

In conclusion, administration of 1000 mg/kg body weight of 70% ethanol extract of *S. guineense* leaf delayed fetal development as witnessed by reduced CRL of 20-day old rat fetuses. High dose treatment with *S. guineense* extract also decreased food intake and weight gain of the pregnant rats that showed its toxicity at high dose. Therefore, care should be taken while consuming the *S. guineense* leaf during pregnancy, especially in a high dose. The results of our study would be a benchmark for further investigation of the test plant and for incorporating the plant into modern pharmaceutical products. Further studies should be conducted by administering the plant extract during the whole period of gestation and isolating secondary metabolites of *S. guineense* leaf with serum level determination to identify the toxic nature and mechanism of action. Based on these findings, it is also advisable to investigate the clinical applicability of *S. guineense.*

## Figures and Tables

**Table 1:** Maternal weight gain and food intake of pregnant rats treated with 70% ethanol leaf extract of *Syzygium guineense*.

Weight gain and food intake	Group				
	Group I 250 mg/kg	Group II 500 mg/kg	Group III 1000 mg/kg	Group IV control	Group V *Ad- libitum* control
Food intake/day (g) *n* = 10	199.2 ± 4.5	191.4 ± 8.0 ^*∗∗*^	180.0 ± 12.1 ^*∗*^!	196.6 ± 10.0	214.0 ± 4.5 ^*∗*^
Maternal weight gain/dam (g)					
Day 6–12	17.8 ± 8.5	17.2 ± 7.4	15.8 ± 7.5	19.0 ± 12.5	40.2 ± 5.8 ^*∗*^
Day 13–20	69.7 ± 3.4	67.2 ± 6.6	60.5 ± 5.6 ^*∗∗∗*^	72.2 ± 5.6	72.8 ± 9.6

The results are expressed as mean ± SDM,  ^*∗*^significant difference with all the other groups,  ^*∗*^! significant difference with Group I, IV and V,  ^*∗∗*^significant difference with group V,  ^*∗∗∗*^significant difference with group IV and V (for all *p* value <0.05); and one-way ANOVA.

**Table 2:** Pregnancy outcomes of rats treated with 70% ethanol leaf extract of *Syzygium guineense*.

Variables	Group				
	250 mg/kg *n* = 10	500 mg/kg *n* = 10	1000 mg/kg *n* = 10	Control *n* = 10	*Ad libitum* control *n* = 10
Number of fetuses	93	91	88	95	115
Number of implantation sites/litter	9.8 ± 2.1	10.0 ± 2.5	10.0 ± 1.4	10.5 ± 0.5	11.7 ± 1.2
Number of resorption sites/litter	0.5 ± 0.8	0.9 ± 0.9	1.0 ± 0.9	1.0 ± 1.6	0.2 ± 0.4
Live fetuses/litter	9.3 ± 2.7	9.1 ± 3.5	8.8 ± 1.6	9.5 ± 1.9	11.5 ± 1.4
Dead fetuses/litter	0	0	0.2 ± 0.4	0	0
Number of male fetuses/dam	4.5 ± 1.6	4.3 ± 1.2	3.7 ± 1.5	5.0 ± 1.1	5.3 ± 1.2
Number of female fetuses/dam	4.8 ± 2.7	4.8 ± 2.4	5.3 ± 2.3	4.5 ± 2.4	6.2 ± 0.8

The results are expressed as mean ± SDM, One-Way ANOVA; *n*: number of dams.

**Table 3:** Fetal growth indices of rat fetuses following the administration of 70% ethanol leaf extract of *Syzygium guineense*.

Variables	Group				
	250 mg/kg *n* = 10	500 mg/kg *n* = 10	1000 mg/kg *n* = 10	Control *n* = 10	*Ad libitum* control *n* = 10
CRL/fetus (cm)	5.3 ± 0.2	5.2 ± 0.4	5.0 ± 0.4 ^*∗*^	5.5 ± 0.2	5.7 ± 0.4
Fetal weight (g)	5.8 ± 0.9	5.1 ± 0.5	4.9 ± 0.5	5.5 ± 0.6	5.1 ± 0.8
Placental weight (g)	0.7 ± 0.2	0.6 ± 0.1	0.6 ± 0.1	0.7 ± 0.1	0.6 ± 0.05

The results are expressed as mean ± SDM,  ^*∗*^significant difference with control and *ad libitum* control groups (*p* value <0.05), One-Way ANOVA; CRL: Crown-rump length.

**Table 4:** Microscopic placental abnormalities of rats following administration of 70% ethanol leaf extracts of *Syzygium guineense*.

Group	Percent of placental abnormalities		
	Decidual cystic degeneration	Hemorrhage/hematoma	Trophoblast proliferation
Group I (250 mg/kg)	0	0	0
Group II (500 mg/kg)	10	10	10
Group III (1000 mg/kg)	20	0	10
Group IV (control)	10	10	0
Group V (*Ad libitum* control)	0	0	0

Results are expressed as percentage of placental abnormalities, Chi-square.

## Data Availability

All data are included in the manuscript.

## Abbreviations

ANOVA: One-way analysis of variance
CRL: Crown-rump length
DPX: Dibutylphthalate polystyrene xylene
EPHI: Ethiopian public health institute
OECD: Organization for economic co-operation and development
SDM: Standard deviation of mean
SPSS: Statistical package for social science.
